# Role of Nuclear Sentinel Lymph Node Mapping Compared to New Alternative Imaging Methods

**DOI:** 10.3390/jpm13081219

**Published:** 2023-07-31

**Authors:** Vincenzo Cuccurullo, Marco Rapa, Barbara Catalfamo, Giuseppe Lucio Cascini

**Affiliations:** 1 Department of Precision Medicine, Università della Campania “Luigi Vanvitelli”, 80138 Napoli, Italy; 2 Nuclear Medicine Unit, Department of Diagnostic Imaging, Magna Graecia University of Catanzaro, 88100 Catanzaro, Italycascini@unicz.it (G.L.C.)

**Keywords:** sentinel node biopsy, lymphatic mapping, lymphoscintigraphy, optoacoustic imaging, fluorescence, radiomics

## Abstract

With the emergence of sentinel node technology, many patients can be staged histopathologically using lymphatic mapping and selective lymphadenectomy. Structural imaging by using US, CT and MR permits precise measurement of lymph node volume, which is strongly associated with neoplastic involvement. Sentinel lymph node detection has been an ideal field of application for nuclear medicine because anatomical data fails to represent the close connections between the lymphatic system and regional lymph nodes, or, more specifically, to identify the first draining lymph node. Hybrid imaging has demonstrated higher accuracy than standard imaging in SLN visualization on images, but it did not change in terms of surgical detection. New alternatives without ionizing radiations are emerging now from “non-radiological” fields, such as ophthalmology and dermatology, where fluorescence or opto-acoustic imaging, for example, are widely used. In this paper, we will analyze the advantages and limits of the main innovative methods in sentinel lymph node detection, including innovations in lymphoscintigraphy techniques that persist as the gold standard to date.

## 1. Introduction

In vivo detection of transport and accumulation of neoplastic cells in the nearest loco-regional lymph nodes has been one of the biggest challenges of modern oncology and diagnostic imaging in the past 50 years [1].

The basic concept is simple: We know that breast cancer cells, for example, move into regional lymph nodes following a sequential pathway [2]. Accordingly,, the lymph node closer to the tumor is metastasized by neoplastic cells in all patients with regional positive nodes [3]. The goal is to identify in vivo the first lymph node draining the anatomical district of the tumor, called the sentinel lymph node. Subsequently, we analyze the lymph node under a microscope or with modern real-time methods, such as nucleic acid amplification (OSNA), to recognize the presence of tumor cells [4]. We can assume that all regional lymph nodes are spared in the case of the inviolate sentinel lymph node; then lymphadenectomy may be avoided, allowing for an enormous cosmetic and functional benefit and preventing surgical complications, such as limb lymphedema. Conversely, a selective or wide lymphadenectomy must be performed when tumor invasion is detected in the sentinel lymph node [5].

In vivo, tracing biological function in real time has always been pivotal for nuclear medicine procedures, where “radiotracers” and not “contrast agents” are used to enrich pure anatomy with metabolism and function [6,7].

However, the role of nuclear medicine could be reconsidered thanks to advancements in anatomical techniques for superficial and deep lymphatic drainage. Upon a detailed review of the scientific literature, it becomes clear that this “potential threat” has to be considered as an addition to the diagnostic scenario. Innovative approaches to this setting concern different biomedical techniques, including new specific radiotracers as well as new signal detection methods, increasing in both detection rate and anatomical definition [8].

Nevertheless, the best results may be reached by combining non-nuclear and nuclear techniques as an optimal equilibrium between the pros and cons of all the procedures used in this setting.

In real practice, a new technique becomes clinical if it unequivocally demonstrates superior results and is cost effective with respect to the nuclear technique, considered the gold standard on the basis of its diagnostic performance and low cost.

The sentinel lymph node mapping is applied to different types of cancer, such as melanoma; head and neck squamous cell cancer; genital carcinomas, such as penis and vulva cancer; gynaecological malignancies, such as cervical cancer and endometrial cancer; and in breast cancer, where it is frequently used [9].

Sentinel lymph node detection has been an ideal field of application for nuclear medicine because anatomical data fails to represent the close connections between the lymphatic system and the regional lymph nodes, or, more specifically, to identify the first draining lymph node. In order to simplify use and to reduce radiation in patients, technicians and surgeons, alternatives to lymphoscintigraphy are now considered. Some of these are preclinical and detect metastases in the sentinel lymph node through optical coherence tomography (OCT).

For example, a recent study by Yang H. of 158 patients has obtained encouraging results, especially using dynamic cell imaging (DCI) in comparison to full-field optical coherence tomography (FFOCT), reaching a specificity of 98.9% [10].

New alternatives without ionizing radiation are now emerging from “non-radiological” fields, such as ophthalmology and dermatology, where fluorescence or opto-acoustic imaging, for example, are widely used [11]. In this review, we will analyze the advantages and limits of the main innovative methods in sentinel lymph node detection, including innovations in lymphoscintigraphy techniques that persists as the gold standard to date in almost all types of tumors. Although recent guidelines provide alternatives.

## 2. Innovative Methods in Clinical Imaging

All the components of the lymphatic system are well assessed via conventional imaging methods in terms of anatomic structure and dimensions. Structural imaging by using US, CT and MR permits precise measurement of lymph node volume, which is strongly associated with neoplastic involvement; the clinical scenario changes when neoplastic spreading in the lymphatic system has to be excluded in patients with a low pre-test probability. On the contrary, an enlarged lymph node is not always due to metastases.

In practice, the optimal imaging technique warrants high positive predictive value in the case of enlarged lymph nodes, and high negative predictive value in normal-volume lymph nodes. Consequently, a positive sentinel node may present irrespective of dimensions; furthermore, effective imaging should be focused on cells’ detection more than the volume and structure of nodes.

Contrast agents in MRI and US (CEUS) as well as opto-acoustic and fluorescence imaging may improve some drawbacks in sentinel node biopsy through traditional diagnostic imaging [12].

### 2.1. MRI

Over the years, MRI has managed to increase its anatomical definition thanks to high-field machines without significant improvements in sentinel lymph node detection. Magnetic resonance lymphangiography can be executed on a 1.5 T or 3.0 T unit; the need for a paramagnetic contrast agent and the unavailability of intraoperative methods for sampling are major drawbacks to address this issue [13]. Nevertheless, some experiences support the integrative use of MRI in conjunction with lymphoscintigraphy [14].

New dedicated MRI sequences, high resolution diffuse-weighted imaging (DWI), spectroscopy and radiomics analyses represent different efforts to use MRI free from contrast agents. The goal of the contrast agent is to change the relaxation time, allowing for the identification of the tumoral node. The most widely used contrast agent is gadolinium. But superparamagnetic iron oxide nanoparticles (SPION) have aroused particular interest; they accumulate in a normal lymph node via deposition into the macrophages, resulting in a loss of signal where the cells are normal [15]. Sites of neoplastic invasions within the node appear as persisting areas of signal through imaging or magnetic probes on the surgical field [16].

Liu et al.’s meta-analysis evaluated the effectiveness of SPION in evaluating sentinel lymph nodes in breast cancer compared to the standard method. The meta-analysis involved 2298 patients from 19 studies and demonstrated that the use of SPION led to 90.0% accuracy in locating sentinel lymph nodes, which was superior to the standard method’s 85.7%. Metastasis was confirmed in 639 sentinel lymph nodes, with corresponding rates of 96.7% for positive sentinel lymph node detected via SPION and 93.9% for those detected with a standard technique [17].

Another meta-analysis, conducted by Karakatsanis A. et al., aimed to compare the diagnostic accuracy of SPION for sentinel lymph node (SLN) biopsy in patients with breast cancer with the gold standard technique (Technetium and patent blue). The meta-analysis of the multi-center prospective study, which included seven eligible studies with a total of 206 patients with early breast cancer, demonstrated similar detection rates of 97.1% with the standard technique and 97.6% with SPION. Similar results demonstrated a detection rate per node of 91.3% with the standard technique and 93.3% with SPION. In 54 patients with determined metastasis, the standard technique and SPION method demonstrated detection rates of 98.1% and 96.3%, respectively, with a concordance rate of 98.1%. The study concluded that the use of SPION in sentinel lymph node biopsy for breast cancer had high diagnostic accuracy compared to the standard technique and could serve as a promising alternative to conventional methods [18].

In the prospective, multicenter UK SentiMag trial (SMART study), conducted in five centers in the UK on a total of 109 cN0 patients, Vydia R. et al. evaluated the diagnostic accuracy of the magnetic technique with SPION and compared it to the gold standard scintigraphic technique. All the patients were evaluated with both techniques. The results demonstrated a detection rate per patient of 98.13% when using the magnetic tracer and 92.26% when using the standard technique. The lymph node detection rate was 93.07% with the magnetic tracer and 95.53% with the standard technique. Both methods were able to identify 100% of the lymph nodes found to be pathological. The study demonstrates a non-inferiority of the method using superparamagnetic iron oxide (SPION) to the radioisotope technique, thus offering a viable alternative to the latter [19].

In recent years, however, an easy-to-use, handheld portable magnetic probe capable of localizing the sentinel lymph node during surgery has appeared in clinical practice. This probe identifies the same contrast agent, previously used in the pre-operative time with MRI, 20 min before surgery-based SPION. This allows for the elimination of ionizing radiation while maintaining a diagnostic accuracy rate almost identical to that of lymphoscintigraphy, which also shows possible artifacts [20].

In a multicenter clinical trial, Taruno K. et al. evaluated in 208 patients the sentinel lymph node detection rate using the newly handheld portable probe, and compared their evaluation to that using the dual technique gold standard. In all the patients, both the radioactive colloids and magnetic tracers SPION were administered in subcutaneous tissue up to 24 h before surgery. The blue dye was administered just before the sentinel lymph node mapping, performed first with magnetic probe and then with gamma probe. The detection rate with the magnetic method and scintigraphic method were 94.8% and 98.1%, respectively. The concordance between the two methods was 96.1%. This study, as well as other studies (Central-Europe SentiMag trial, SentiMag multi central trial), demonstrates the non-inferiority of the magnetic method using magnetic probe to the scintigraphic method in sentinel lymph node mapping [21].

MRI is a safe and effective tool also during pregnancy, as it has excellent spatial and contrast resolution, is free of ionizing radiation, and yields non-operator-dependent results [22].

### 2.2. CEUS

Contrast-enhanced ultrasound (CEUS), like other diagnostic methods, also uses a contrast agent in order to identify the first lymph node that accumulates it in real time [23]. The contrast agents used are divided according to the rigidity of the outer envelope, which contains an inert gas, usually sulfur hexafluoride, inside it [24]. The outer shells can be made of polymers, proteins or lipids that affect their performance [23].

In a recent study by Machado P. et al., the use of CEUS was compared with the gold standard method of dual techniques. Ultrasound contrast agent, blue dye and radiotracer were administered to the 79 patients with early-stage breast cancer. A similar accuracy was observed in the use of an ultrasound contrast agent compared with the use of a radiotracer (99.4% vs. 96.2%), and superiority in the accuracy of CEUS compared with the use of a blue dye alone (86.5% vs. 68.5%) was observed. Of the 252 lymph nodes evaluated, 223 were positive for ultrasound contrast agent, and all 34 malignant lymph nodes evaluated subsequent to histology were positive for CEUS [25].

The advantages of being able to identify the sentinel lymph node in only one preoperative time are considerable in this technique; in fact, the method uses instruments that are easily transportable and quickly applied, and it avoids the use of ionizing radiation. Several disadvantages limit its application, such as low spatial resolution and instability of contrast bubbles. However, the main disadvantage is the dependence of the result based on the operator’s qualifications, with long learning times and the need for dedicated staff in the pre-operative phase. Of interest is the development of contrast agents for therapeutic purposes that are under evaluation in preclinical studies.

### 2.3. Fluorescence Method

Fluorescence imaging has made its appearance in the area of the sentinel lymph node detection for just over a decade. Its wider use involves other areas of medicine, particularly ophthalmology, where it plays a central role in retinal fluoroangiography [26]. Other modern fields of application include intraoperative imaging for the identification of intestinal blood vessels and in the surgery of liver lesions. Fluorescence imaging exploits the ability of a contrast agent (fluorophore), such as indocyanine green (ICG), to absorb light emitted with a wavelength of 760 nm and re-emit it at a higher wavelength of 820–830 nm on the near infrared (NIR) spectrum. A NIR camera positioned on the study field identifies the emission in infrared spectrum, which is invisible to the human eye [27].

In a prospective observational study, Dumitru D. et al. evaluated in 79 patients the accuracy of ICG compared with radioisotope for sentinel lymph node detection. The study demonstrated a procedural detection rate of 97.5% for radioisotopes and 98.7% for indocyanine green, with a better nodal detection rate for indocyanine green compared with radioisotopes (98.1% vs. 73.4%). All the positive metastatic nodes were detected through both CEUS and radioactive techniques. This study confirms the non-inferiority of the fluorescence method compared to the radioisotope method compared to the “gold standard” radioisotopic method [28].

Similar results were found in a study by Samorani D. et al., one of the largest in terms of the number of patients involved. This prospective study evaluated in 301 patients the detection rate of the sentinel lymph node by comparing the 99m-Tc nanocolloid method and the indocyanine green fluorescence method. The results demonstrated a lymph node detection rate of 99% with the indocyanine green method and 76.7% with the 99m-Tc nanocolloid method alone, and a concordance rate of 98.75%, suggesting that the fluorescence method could be validated as an alternative method to 99m-Tc SLN detection in breast cancer [29].

The opportunity to identify the sentinel lymph node in real time, with the possibility of increased resolution through microscopic methods, combined with the safety of the ICG, renders the method extremely promising, even in combination with other contrast agents. The main disadvantage that remains is the method’s poor tissue penetration, which does not exceed 12 mm, that could be reduced in the coming years by using multimodal methods with scintigraphic imaging or nanomaterials with better fluorescence characteristics than ICG. These nanomaterials, such as quantum dots or carbon nanoparticles, absorb light in the traditional NIR 1 wavelength window, but emit a broader-spectrum, higher-power infrared signal in the NIR 2 wavelength window (1000–1700 nm), with the advantage of being able to improve both resolution and deep tissue penetration. In recent years, more specific molecules marking lymphatic structures have been studied; these not only make it possible to selectively identify only certain tumor types, but also make it possible to selectively identify lymphatic vessels or tumor margins. These molecules, instead of accumulating specifically like current contrast agents, are able to selectively bind membrane proteins or insert themselves into specific enzymatic pathways, which in turn causes them to become activated. These molecules usually consist of monoclonal antibodies already in therapeutic use, such as those against VEGF (bevacizumab), EGFR (cetuximab) or HER2 (trastuzumab), but conjugated to an emitting fluorophore in the NIR spectrum. The concept is the same as modern theragnostic, in which it is possible to identify in vivo using an NIR camera the localization of conjugated molecules while knowing that the latter also have a therapeutic effect. Currently, these molecules have not yet been studied in humans, but the preclinical results look extremely promising. Within the scope of fluorescence methods also fall the Cerenkov effect. More precisely, Cerenkov luminescence is light generated when charged particles exceed the speed of light in a dielectric medium. The Cerenkov effect is given by radioisotopes used in PET imaging such as 18-F, thus finding practical clinical application [30].

### 2.4. Optoacoustic Imaging

One of the most promising methods for the future is optoacoustic imaging [31]. This recent hybrid method uses the detection of ultrasonic waves by a probe generated through thermoelastic expansion as a result of laser-induced emission [32]. In this multispectral method, several vascular structures can be identified simultaneously, differentiating the arterial from the venous compartment in relation to the different amount of oxyhemoglobin and deoxyhemoglobin [33]. More difficult is identifying the lymphatic structures and, in particular, the sentinel lymph node without an adequate contrast agent. The optimal contrast agent must accumulate in the first capturing lymph node and allow a return signal to be generated that provides an optimal signal-to-noise ratio (SNR) [34]. Colloidal nanobeacons, which contain gold nanoparticles with an excellent safety profile and strong lymph node retention, permit an improvement in SNR. Others could be contrast agents already used in other methods, such as blue dyes and ICG, which could make the combined method more sensitive. The optoacoustic method of the sentinel lymph node in melanoma is interesting because of the peculiar properties of melanin accumulated in cancer cells that render it a true natural contrast agent [35]. In melanoma, the concordance rate between multi spectral optoacoustic tomography plus the indocyanine green fluorescence method and lymphoscintigraphic plus single-photon emission tomography (SPECT) was evaluated in the cross-sectional study by Stoffels I. et al. The results demonstrated in 83 patients, in whom a total of 165 sentinel lymph nodes were evaluated intraoperatively, a concordance rate between the two methods of 96.4%. The false negative sentinel lymph node rate was 0% in both methods. This study demonstrates that the non-radioactive method via optoacoustic imaging allows for the identification of the sentinel lymph node at a frequency equivalent to that of the current radiotracer conventional standard. To date, a similar comparison study in the field of sentinel lymph node detection in breast cancer is lacking. The optoacoustic method represents the one with the greatest potential for growth in the coming years as a complementary method to other scintigraphic and fluorescence methods, with its main limitations being its low specificity and operator-dependent results [36].

At a later stage, it could assume a “theragnostic” role (both diagnostic and therapeutic). In diagnostic terms, thanks to the spiral volumetric optoacoustic tomography method, which allows for three-dimensional (3D) images with very high spatial and temporal resolution, the method is capable of assessing the effects of neoadjuvant chemotherapy, guiding surgical resection and monitoring drug delivery [37]. In therapeutic terms, using photothermal and photodynamic agents belonging to several families of molecules that are activated via laser emission will act mostly at the vascular level by reducing the supply of oxygen and nutrients to the tumor [38]. Another therapeutic example is the possibility of conjugating chemotherapeutics into nanoparticles activated in situ via laser emission, such as doxorubicin, which can be inserted into fucoidan-capped gold nanoparticles that are destroyed via laser emission once they reach the tumor level [39]. Currently, the use of nanoparticle therapies and photosensitive drugs are being evaluated in phase 1 studies.

### 2.5. Radiomics

Artificial intelligence (AI), and, more specifically, machine learning, is quietly landing in imaging methods thanks to the application of modern neural networks [40]. Although it currently represents only a potential application in the distant future, it promises to achieve incredible results through the early identification of lymph node metastases on radiological imaging that would avoid sentinel lymph node biopsy. One of the applications of AI is in the field of radiomics applied to MRI images [41].

A recent retrospective study by Yu Y. et al. evaluated the feasibility of using dynamic contrast-enhanced magnetic resonance imaging (DCE-MRI) radiomic signature in a noninvasive assessment of lymph node status in patients with early-stage breast cancer. The study evaluated a total of 1214 women divided into two groups, and it validated clinical-radiomic nomograms that could predict the presence of axillary lymph node metastasis and 3-year disease-free survival by discriminating patients at high and low risk of recurrence. Clinical-radiomic decision curves were demonstrated to be superior to single curves alone. The study demonstrated a real application of using MRI-based machine learning in decision-making optimal treatment in patients with early-stage breast cancer [42].

A recent study by Haraguchi T. et al. demonstrated the potential of noninvasive determination of the sentinel lymph node in 100 patients with breast cancer and a histologically proven cN0 parameter. It was observed that the use of machine learning models using the radiomics feature from diffusion-weighted whole-body imaging with background signal suppression (DWIBS) sequence and short tau inversion recovery (STIR) demonstrated potential use in biopsy-free evaluation on sentinel lymph node status, and encouraged new studies on its use in clinical practice [43].

Encouraging results come from a study by Guo X. et al. who developed and validated a predictive model using images obtained with ultrasound and combining them with deep learning radiomics and axillary ultrasound. This model aimed to predict the risk of metastasis at the sentinel lymph node and the other axillary lymph nodes evaluated. This model achieved a sensitivity performance of 98.4% in both identifying patients with metastasis in the sentinel lymph node and other lymph nodes. The model also accurately stratified lymph node metastasis-negative patients into low-risk and high-risk groups by achieving a negative predictive value (NPV) of 97% and 91.7%, respectively [44].

## 3. Lymphoscintigraphy and New Updates in a Gold Standard Technique

Lymphoscintigraphy represents the gold standard method in the sentinel lymph node biopsy to date, with a detection rate higher than 95% [45]. The standard procedure establishes the peritumoral administration of vital blue dye and 99m-Tc labelled nanocolloids the day before surgery (dual technique) [46].

The surgeon uses a gamma probe to detect the radioactivity emitting from lymph node and removes it along with blue colored nodes, even though they do not always match each other [47].

99m-Tc is the isotope of reference according to optimal physical properties. The different molecules targeted with 99m-Tc and relative pharmacological properties are discussed below. More recently, radionuclides emitting positrons, such as 18-F and 68-Ga, have been introduced to take advantage of the biological value of targeted pharma and spatial resolution [48,49,50].

The differences among 99m-Tc-labelled radiotracers are due to their particle sizes and surface charge: small molecules (<10 nm) pass more rapidly into lymphatic vessels with lower node retention and are used in the lymphographic study. The larger nanoparticles are trapped in the lymph node with a higher persistence throughout time; thus, they are ideal for sentinel lymph node detection [51].

The optimal size of a molecule for the sentinel lymph node has not been defined yet, but an acceptable cut off is considered >40 nm [52].

One of the first studies to identify the optimal size of nonacolloids was performed by De Cicco C. et al. The authors compared three different ranges in size of 99m-Tc-labeled colloid particles in 250 patients; they obtained the best detection rate (96%) by using a 200–1000 nm colloid, administered sub-dermally in an injection volume of 0.4 mL [53].

Vermeulen K. et al. have reviewed several types of nanocolloids with overlapping characteristics, reporting a wider use of 99m-Tc nanocolloid of human serum albumin. In recent years, new 99m-Tc radiotracers have been introduced with encouraging results, reaching a detection rate of 100% and a very low false negatives ratio [51].

The 99m-Tc Tilmanocept (lymphoseek) and 99m-Tc rituximab emerge among these new promising tracers.

The 99m-Tc Tilmanocept consists of a dextran backbone to which multiple units of mannose and DTPA are added [54]. The sugar selectively binds CD206 expressed by macrophages and dendritic cells at the lymph node level [55]. The greater selectivity of Tilmanocept [56], due to increased retention in the lymph node compared with nanocolloids, [57] allows not only for a lower radiation dose administered to the patient but also for higher detection rates [58].

Tokin et al. estimated the accuracy of Tilmanocept by performing a comparative review and a subsequent meta-analysis. They collected five reviews demonstrating a detection rate of 95.91% by using 99m-Tc nanocolloid; this satisfactory result was lower than 99.99%, which was obtained during a phase 3 study for the registration of 99m-Tc Tilmanocept. This molecule presents lower pain at the injection site, and it permits multi-modal imaging associated with NIR fluorescence dye [59].

99m-Tc rituximab is a monoclonal antibody directed to the surface receptor CD20, which is expressed by B-lymphocytes resident in lymph nodes [60]. The molecule exhibits rapid clearance from the injection site, a higher detection rate than conventional radiotracers, such as tilmanocept and an improved safety profile.

The study of Li N. et al. is one of the most recent retrospective studies that evaluated the accuracy of 99m-Tc rituximab in 533 melanoma patients. The detection rate of the sentinel lymph node was 98.1%. In 90 patients in which regional lymph node dissection was performed after sentinel lymph node biopsy, the results demonstrated a sensitivity, specificity and accuracy of 97.4%, 100% and 96.7%, respectively, in metastatic diagnosis [61].

In any case, the greater diagnostic accuracy achieved using these new agents may lead to a replacement of 99m-Tc nanocolloid in the coming years only if their costs are competitive, as to date they are still too high for national health care systems.

Interesting opportunities arise from combining different 99m-Tc-based tracers with vital blue dyes to reduce the time required by the standard dual technique [62].

Hybrid methods are logical consequences of improving diagnostic accuracy. The effective demonstration of the addiction of properties by using hybrid techniques have been reported with 99m-Tc nanocolloid and indocyanine green (ICG) [63]. The hybrid practice prescribes simultaneously NIR camera and gamma probe use in the operating room, taking advantage of the increased retention of the tracer to detect the sentinel lymph node.

Dell’Oglio et al. have reported an improvement in sensitivity using the hybrid ICG/99m-Tc nanocolloid technique in patients with penile cancer, when compared to 99m-Tc with blue dye; the study enrolled 400 patients to compare the detection rate in the fluorescence group against the standard procedure: they concluded that by adding fluorescence, the rate increased by 39% [64].

Hybrid methods may be performed sequentially in multimodal imaging, as in case of three different molecules (99m-Tc nanocolloid, blue dye and indocyanine green) in the study of lymphatic drainages of the breast and arm. This approach, called triple mapping, has been tested in breast cancer patients to preserve unilateral arm lymphatic drainage [65]. Triple mapping is applied as part of axillary reverse mapping (ARM). This technique was first introduced by Thompson et al. [66], and it is based on the hypothesis of the presence of dual and anatomically distinct interconnected lymphatic drainage pathways, on the ipsilateral affected breast. The physiological drainage of the arm may be recognized with ICG with an NIR camera, and the sentinel lymph node is mapped using the standard technique with blue dye and Tc-nanocolloid, allowing for the preservation of arm drainage during the axillary dissection procedure, hence preventing lymphoedema.

Triple mapping is now considered effective for improving outcomes in breast-cancer related lymphedema, maintaining sentinel lymph node detection rate values identical to those of the dual technique method.

A recent meta-analysis covering five studies between 2012 and 2019, with a total of 1696 patients, was performed by Co M. et al. They demonstrated a clear reduction in the incidence of lymphedema in the ARM group (4.8%) compared to the standard mapping (18.8%). The authors reported that there were no differences in disease recurrence between the two groups [67].

Another meta-analysis on 4954 patients from a total of 29 studies was conducted by Wijaya et al.; their study demonstrated identical results, with a reduced incidence of cancer-related lymphedema in the ARM group (2% vs. 14%). The study also demonstrated that the patients who underwent preoperative chemotherapy had no significant difference in outcome in the ARM technique compared with patients without neoadjuvant chemotherapy. Therefore, this method can be performed in both situations. The method also makes it possible to select patients who can receive a lymphovenous by-pass if the two lymphatic pathways are intrinsically connected after a subsequent lymphadenectomy [68].

Tomographic acquisition by using SPECT and SPECT-CT has been introduced to increase spatial resolution, with good clinical results, but at the expense of more resources and more radiation exposure.

Several studies have demonstrated excellent results for a “portable” alternative to the SPECT method that uses a free hand probe capable of achieving real-time 3D spatial reconstruction mapping. In fact, the miniaturization of the SPECT method enclosed in a probe has made it possible to bring into the operating room an imaging method that otherwise requires a dedicated room [69]. In a retrospective study, Argentou M. et al. compared the use of freehand SPECT 3D with the conventional gamma probe in 200 patients, reporting the detection of at least 3 nodes in 83.3% of the patients vs. 72.0% with the gamma probe [70].

A significant increase in the detection rate was also noted in obese patients when evaluated with freehand SPECT. Moreover, the surgeon quickly learns to localize a tridimensional sentinel lymph node, because the area of interest is reconstructed and displayed in real time. Innovative approaches with augmented reality represent the subsequent step to increase surgical confidence and the detection rate, but they reduce the learning curve [70].

## 4. Advantages and Disadvantages of Nuclear and Non-Nuclear Methods

The advantages and disadvantages of the main methods of sentinel lymph node mapping are peculiar to each individual technique. Starting with the lymphoscintigraphic method, which is the gold standard today, although updated thanks to recently used machines, it demonstrates significant advantages and disadvantages. Starting with the advantages, this method maintains very high sensitivities even today, perhaps one of the main reasons for its constant use since it first appeared in clinical practice several decades ago now. This same advantage has also been the reason for its poor potential for upgrading using newer radiotracers (tilmanocept and tc-rituximab), as only a few of these over time have been demonstrated to be truly considered an upgrade from reliable nanocolloid tracers. Less impactful have been the upgrades in the use of hybrid machines that, while they have added improvement in anatomic definition, this has been relegated to pre-operative nodal assessment. It remains to be seen what the future of the free-hand SPECT method will be, the only upgrade in the operative time of sentinel lymph node mapping, compared to gamma probe that could facilitate the surgeon’s operation, especially if implemented with virtual reality. There is no shortage of disadvantages involving the handling of radiotracers that require a nuclear medicine unit with qualified personnel to perform this method. Although the cost of nanocolloids has been decreasing over time, it remains relatively high. The cost of new radiotracers is a major disadvantage of these, considering that the gain in detection rate is not much higher than that obtained with radiocolloids [71].

Regarding non-nuclear methods, whether their advantages justify their use in combination with or instead of nuclear methods is being assessed. Starting with methods of radiological interest, such as CEUS and MRI, both offer the advantages of not needing a nuclear medicine unit and using contrast agents with low cost, as well as their extreme safety compared to a nuclear method that exposes the patient and health care workers to ionizing radiation. Still for both, however, the sensitivity is lower than for scintigraphic methods, and for this reason they are unlikely to replace the latter in the coming years. More promising, however, are fluorescence and optoacoustic methods. Both have the same advantages of safety and ease of management and use, even promising sensitivities identical to scintigraphic methods. The disadvantages of the fluorescence method are its poor identification of deep lymph nodes—which is easily solved by the combined use of the scintigraphic method—and the high cost of the contrast agent it uses. As for optoacoustics, the main disadvantage is its low sensitivity—but this is a method that is still in an early stage of study.

The following tables summarize the main advantages and disadvantages of the various scintigraphic (Table 1) and non-scintigraphic (Table 2) methods discussed above, and some results from current studies performed on novel sentinel node detection techniques (Table 3).

## 5. Conclusions

Today, modern equipment makes it possible to highlight increasingly smaller lesions, and therefore makes extremely targeted and selective surgery necessary [77]. Nuclear medicine remains the gold standard in sentinel lymph node mapping to date, irrespective of which tracer is used. This is particularly true when performed simultaneously with blue dye.

The robustness of this method is due to its efficacy, safety, large diffusion and simplicity of use that it has largely demonstrated during the last 30 years. A success story cannot be written by a one hand: Surgeons have learned and optimized SLN mapping, transforming the diagnostic procedure with a surgical tool. Radiomic or high-resolution imaging methods have a primary limitation: transporting the spatial references of images to surgical plans on patients’ beds.

The registration process of spatial coordinates from scintigraphy or fluorescence, in anatomical plans of CT imaging, then in the corresponding spatial dominion at the patients’ bed, appear as a double back. The larger availability of hybrid imaging as SPET/CT devices has partially simplified this process because its permits the co-registration of the function and anatomy at the same time, in the space of the patient, and with high precision. However, hybrid imaging has demonstrated higher accuracy than standard imaging in SLN visualization on images, but it did not change in terms of surgical detection.

This gap might be reduced in the future if specific contrast agents become available. Some things are now moving in an augmented reality, with surgical simulators to transpose imaging findings in surgical planes; but a lot of water will pass under the bridge before it might be of clinical value. It is difficult to imagine a new, single diagnostic method able to undermine scintigraphy in SLN mapping.

Little to find out remains in terms of pre-surgical lymph node mapping by using imaging; the large part of the procedure has focused on the diagnostic sensitivity in sentinel node detection, resulting in high accuracies and concordance rates among different methods.

However, the pivotal role of the histological assessment, as part of the SLN procedure, is underestimated: It permits a very significant prediction of nodal status, especially if negative. In practice, SLN mapping by using scintigraphy combines the diagnostic accuracy of lymphoscintigraphy with the negative predictive value of a negative histology. Moreover, this is the sole approach that warrants the recognition of small foci of invasion within the node.

Instead, we have high expectations from the combinations of different procedures in this setting: Infrared fluorescence and optoacoustic imaging combined with nuclear medicine appear to be the correct way to go. The idea is to trace different aspects of the same compartment more than to enforce the diagnostic ability of two separate methods. The aim in breast cancer patients is to prevent lymphedema, over imposing the LNS to the physiological lymphatic way. This approach, at the basis of ARM mapping, is a compromise between oncological safety and prevention of complications.

Certainly, improvement in treatment is reached not only using new techniques or sophisticated computer-guided approaches [78,79], but also combining different clinical competences and new strategies in selected patients.

## Figures and Tables

**Table 1:** The main advantages and disadvantages of the various scintigraphic.

**Lymphoscintigraphy Tecnique**	**Advantages**	**Disadvantages**	**Ref.**
99mTc-nanocolloid	High detection rate (96%) and low false negative ratio (5%) High depth penetration	Radiation exposure Painful injection and relative high cost	[52,72]
99mTc-tilmanocept	Reducing pain during injection Reducing radiation dose	High cost	[59]
99mTc-rituximab	Fast clearance from injection site	High cost	[60]
99mTc/ICG	Long retention in the sentinel lymph node Maximum sensibility	Difficult to using both NIR and gamma probe High cost	[73]
Triple mapping (ARM)	Ability to perform ARM to reduce cancer-associated lymphedema	High cost and long learning curve	[59,72]

**Table 2:** The main advantages and disadvantages of the various non-scintigraphic.

**Novel Technique**	**Advantages**	**Disadvantages**	**Ref.**
MRI	Increased anatomical detail with 3D volumetric imaging No radiation exposure	Low sensitivity and possible artifacts	[60]
SPION probe	High detection rate (97%) and low false negative ratio (4%) Long retention in the lymph nodes Long half life	Reduced transcutaneous signal Allergy risk to contrast agent	[74] [17]
CEUS	Low cost and safety No allergy risk	High false negative ratio and long learning curve	[65]
Fluorescence method	High resolution in real-time No radiation exposure Safety profile Short learning curve	Low detection rate of deeper lymph nodes Short half-life	[14,75,76]
Optoacoustic imaging	Deep tissue imaging in real time and high definition	Low specificity Not yet available	[17]
Radiomics	Ability to generate predictive models that can accurately establish axillary free sentinel lymph node positivity	Heterogeneous models not yet standardized and validated in clinical practice	[42,43,44]

**Table 3:** Current studies performed on novel sentinel node detection tech-niques.

Technique	Studies [Type]	No. Patients	Accuracy/ Detection Rates	Corresponding Rates in Metastasis
MRI with SPIO	Liu et al. [meta-analysis]	2298 patients from 19 studies	90% in SPIO vs. 85.7% in standard technique	96.7% in SPIO vs. 93.9% in standard technique
	Karatkatsanis, A. et al. [meta-analysis]	206 patients from 7 studies	97% in SPIO vs. 97% in standard technique	96% in SPIO vs. 98% in standard technique
	Alvarado, M.D. et al. [prospective trial]	146 patients	99% in SPIO vs. 98% in standard technique	95% in both technique
	Douek, M. et al. [prospective multicenter trial]	160 from 7 center	94% in SPIO vs. 95% in standard technique	92% in SPIO vs. 96% in standard technique
	Houpeau, J.L. et al. [prospective multicenter trial]	108 patients	97% for SPIO vs. 95% for standard technique	97% in SPIO vs. 95% in standard technique
	Vydia, R. et al. [prospective trial]	109 patients in 5 centers	98% in SPIO vs. 92% in standard technique	100% in both techniques
	Taruno, K. et al. [multicenter trial]	208 patients	94.% in SPIO vs. 98% in standard technique	
CEUS	Machado, P. et al.	79 patients	99% in CEUS vs. 96% for radioactive tracer vs. 68% for blue dye	100% in CEUS vs. 68% for radioactive tracer vs. 53% for blue dye
	Liu, J. et al.	75 patients	71%	98% in CEUS
Fluorescence method	Dumitru, D. et al. [prospective observational study]	79 patients	98% for indocyanine green vs. 97% for radioisotopes	100% in both techniques
	Samorani, D. et al. [prospective observational study]	301 patients	99% for indocyanine green vs. 76% for radioisotope	
	Agrawal, S.K. et al.	207 patients	97% for indocyanine green plus methylene blu vs. 95% for standard technique	
	Somashekhar, S.P. et al.	100 patients	96% for indocyanine green vs 94% for standard technique	
	Wishart, G.C. et al.	99 patients	100% for indocyanine green vs. 73% in standard technique	
Optoacoustic imaging	Stoffels, I. et al. [cross sectional study]	83 patients	94% with optoacoustic vs 100% with lymphoscimtigraphy	
